# Systematic Analysis of 4-gene Prognostic Signature in Patients with Diffuse Gliomas Based on Gene Expression Profiles

**DOI:** 10.7150/jca.54565

**Published:** 2021-05-19

**Authors:** Chunhai Huang, Zujian Xiong, Qi Yang, Xuejun Li

**Affiliations:** 1 Department of Neurosurgery, First Affiliated Hospital of Jishou University, Jishou, Hunan, 416000, China.; 2 Centre for Clinical and Translational Medicine Research, Jishou University, Jishou, Hunan, 416000, China.; 3 Department of Neurosurgery, Xiangya Hospital, Central South University, Changsha, Hunan, 410008, China.; 4 Xiangya School of Medicine, Central South University, Changsha, Hunan, 410008, China.; 5 Hunan International Scientific and Technological Cooperation Base of Brain Tumor Research, Xiangya Hospital, Central South University, Changsha, Hunan, 410008, P. R. China.

**Keywords:** diffuse glioma, prognosis model, expression data, bioinformatics, systematic analysis

## Abstract

**Background:** Diffuse gliomas are a group of diseases that contain different degrees of malignancy and complex heterogeneity. Previous studies proposed biomarkers for certain grades of gliomas, but few of them have conducted a systematic analysis of different grades to search for molecular markers.

**Methods:** WGCNA was used to find significant genes associated with malignant progression of diffuse glioma in TCGA glioma sequencing expression data and the GEO expression profile-merge meta dataset. Lasso regression was used for potential model building and the best model was selected by CPE, IDI, and C_index. Risk score model was used to evaluate the gene signature prognostic power. Multi-omics data, including CNV, methylation, clinical traits, and mutation, were used for model evaluation.

**Results:** We found out 67 genes significantly associated with malignant progression of diffuse glioma by WGCNA. Next, we established a new 4 gene molecular marker (KDELR2, EMP3, TIMP1, and TAGLN2). Multivariate cox analysis identified the risk score of the 4 genes as an independent predictor of prognosis in patients with diffuse gliomas, and its predictive power was independent of the histopathological grades of glioma. Further, we had confirmed in five independent test datasets and the risk score remained good predictive power. The combination of the prognosis model with specific molecular characteristics possessed a better predictive power. Furthermore, we divided the low-risk group into three subtypes: LowRisk_IDH1^wt^, LowRisk_IDH1^mut^/ATRX^mut^, and LowRisk_IDH1^mut^/ATRX^wt^ by combining IDH1 mutation with ATRX mutation, which possessed obvious survival difference. In further analysis, we found that the 4 gene prognosis model possessed multi-omics features.

**Conclusion:** We established a malignant-related 4-gene molecular marker by glioma expression profile data from multiple microarrays and sequencing data. The four markers had good predictive power on the overall survival of glioma patients and were associated with gliomas' clinical and genetic backgrounds, including clinical features, gene mutation, methylation, CNV, signal pathways.

## Introduction

Gliomas are the most common and lethal type of primary CNS tumors in adults. Among them, diffuse gliomas (DGs) constitute the majority of gliomas, including lower-grade gliomas (LGG, grade II-III), and glioblastomas (GBM, grade IV) according to WHO classification 1. Due to intra- and inter-tumoral heterogeneity, gliomas' molecular characteristics, and clinical phenotypes could be different, even if identified as the same histological grade. For example, grade II glioma with IDH mutation and 1p19q codeletion possesses a better prognosis, whose median survival is 96 months, than the one with IDH wild type whose median survival is merely 20.4 months-long 2. Although regarded as benign-natured tumors, more than 70% of diffuse LGGs would transform into anaplastic gliomas or even secondary glioblastomas in 10 years 3. The classification system based on histology is not sufficient for glioma precise diagnosis, thus WHO introduced molecular characteristics into glioma classification system in 2016 for improving diagnostic accuracy. Meanwhile, progress in the development of genome sequencing technologies and bioinformatics tools shed a light for us on understanding the pathology of gliomas. By analyzing these sequencing data, researchers have found plenty of specific molecular markers like IDH mutation, MGMT promoter methylation, and TERT mutation, etc. These molecular markers of glioma gave us a novel understanding of the mechanism and diagnosis of glioma, and introduced new treatments for glioma patients 4, 5. However, the molecular mechanisms of different survival outcomes and tumor heterogeneity are still unclear and remain further exploration. For improving the diagnosis accuracy and precision of prognosis prediction, it is necessary for us to search for more effective molecular markers and targets.

Previous studies have confirmed that tumor classification based on expression profiles is an objective and reliable method to help diagnosis and treatment, and lots of practices were performed on glioma by now 6. But most of them were inclined to a specific glioma subtype, there are few studies build an adequate prediction model for all DGs. In our study, we united multiple complete large-scale gene expression profiles, high-throughput sequencing datasets and multidimensional data, including gene mutations, copy number variations and methylation datasets, of DGs to figure out the most responsible molecular markers for glioma prognosis.

## Materials and methods

### Data collection

We obtained TCGA glioma RNA-seq raw count data, copy number variation (CNV) data, DNA methylation data, mutation data, and corresponding clinical information from GDC by using R package *TCGAbiolinks*. RNA-seq raw count data were preprocessed and normalized with R package *DESeq2* and R package *preprocessCore*. CGGA RNA-seq data and microarray data (level 3) is obtained from http://cgga.org.cn. Besides, we obtained 9 datasets from the GEO database (https://www.ncbi.nlm.nih.gov/geo) (Tab.S1). All files obtained from the GEO datasets were in the CEL format and were preprocessed by R package *affy* and *gcrma*. Due to limited patient size or incomplete pathological information, we merged 6 microarray sets (GSE43378, GSE19728, GSE4290, GSE61374, GSE43289, GSE7696) in the Hg-u133 Plus 2.0 platform into one large dataset (named meta_GSE1) and corrected batch effect by using R package *sva*. The batch effect in the CGGA RNA-seq data was also removed. Another 3 GEO datasets (GSE16011, GSE68848, GSE74187) were downloaded for model validation after batch effect removal. All non-DGs samples from all datasets were excluded from this study. We merged the gene symbol with multi probes and the average value was used. The study was approved by the Jishou university ethics review committee.

### Identification of tumor progression-associated genes with weighted correlation network analysis (WGCNA)

In order to screen progression-associate genes, we used the expression profile data from TCGA and meta_GSE1 and constructed a co-expression network using R package *WGCNA* 7. Common genes in the most grade-relevant module identified from these two datasets respectively were selected as the candidate for tumor progression-associated genes. Briefly, we first filtered out genes with missing value or low variable genes (var < 0.05). Outliers in samples were detected and removed. Next, we calculated s_ij_ and the adjacency matrix a_ij_ as follows: s_ij_ = |cor(x_i_, x_j_)|, a_ij_ = S_ij_^β^. Where X_i_ and X_j_ were vectors of expression value for gene i and j, s_ij_ represented the Pearson's correlation coefficient of gene i and gene j, a_ij_ encoded the network connection strength between gene i and gene j 8. The power of β = 9 (scale free R^2^ = 0.95) was selected as the soft-thresholding parameter to ensure a scale-free network. Then, average linkage hierarchical clustering was conducted based on a topological overlap matrix (TOM)-based dissimilarity measure. We considered 30 as the minimum number of genes in each module. The gene modules were identified using the DynamicTreeCut algorithm and merged with a cut-height of 0.2. The module eigengenes (MEs) were generated as the first principal component after the principal component analysis was performed with the expression data for co-expressed modules. Module membership assignment (kME) was determined as Pearson's correlation coefficient between gene expression values and MEs. The candidate tumor progression-associated genes were screened from modules highly correlated with glioma grade (Absolute value of gene significance (GS) ≥0.2 and module membership (MM) absolute value ≥0.8).

### Prognostic model construction

First, we screened genes that are associated with prognosis by univariable Cox analysis using coxph function of R package* survival* from candidate tumor progression-associated genes. To identify common prognosis-associated genes regardless of glioma grades, we selected intersected genes from all survival-associated genes in patients with grade II, III, and IV DGs separately (p < 0.05). To construct a robust model, we excluded samples with a survival time of less than one month as these patients may die due to reasons other than tumors. 630 samples were obtained for further analysis. To reduce overfitting, we used the Least Absolute Shrinkage and Selection Operator (LASSO) regression analysis to filter these genes by R package *glmnet* 9, 10*.* The best lambda value is selected by the *cv.glmnet* function. The predictive ability of the model was determined by C_index, Concordance Probability Estimate (CPE), and integrated discrimination improvement (IDI) method (using coxph function, R package *clinfun*, and R package *survIDINRI*, respectively). A risk score model was constructed based on the expression levels of prognosis-associated genes and the contribution coefficient (β) of the univariate Cox proportional hazards regression model.


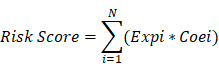


N, Expi, and Coei represented the number of signature genes, gene expression level, and coefficient value, respectively. The risk score was then divided into high and low-risk groups with the optimal cut-off value which calculated by surv_cutpoint function. Multivariate Cox analysis was conducted from the analyse_multivariate function in R package *survivalanalysis*. Prognostic receiver operating characteristic (ROC) curves of risk scores were then created from 1 to 10 years.

### Integrated analysis combined clinical and multi-omics data of risk scoring model

Further, we used TCGA glioma expression profiles data, gene mutations data, CNV, methylation data, and immune activity to investigate the underlying mechanisms in each risk subgroup. Immune gene signatures were obtained from the R package *imsig* (https://github.com/ajitjohnson/imsig). ImSig is a set of gene signatures that can be used to estimate the relative abundance of immune cells in tissue transcriptomics data, especially in cancer datasets. The KEGG gene signatures were obtained from the Molecular Signatures Database v6.2 version (http://software.broadinstitute.org/gsea/downloads.jsp). GSEA analysis was performed by R package *clusterProfiler* 11. Tumor ESTIMATE score, immune score, stromal score, and tumor purity were analyzed with R package *estimate*. Gene mutations data, DNA methylation data, and CNV data were preprocessed and analyzed using R package *TCGAbiolinks*. 101 glioma-related driver genes that we selected were derived from the mutational cancer driver database (https://www.intogen.org/search) 12. Gene mutation analysis was performed by R package *maftools*. The R package *ComplexHeatmap* was used for heatmap drawing. The *GOplot* package was used for Gene Ontology (GO) annotation. The R package *ggstatsplot* was used for correlation analysis and plotting.

### Statistical analysis

All statistical analyses were performed with R (version 3.5.2, http://www.r-project.org). The optimal cut-off value for patients' risk stratification was performed by surv_cutpoint function in R package* survminer.* Survival differences between the low-risk and high-risk groups were assessed by the Kaplan-Meier estimate and compared using the log-rank test. All statistical tests were two-sided and P values < 0.05 were considered statistically significant.

## Results

### Identification of glioma progression related genes

**Figure 1:**
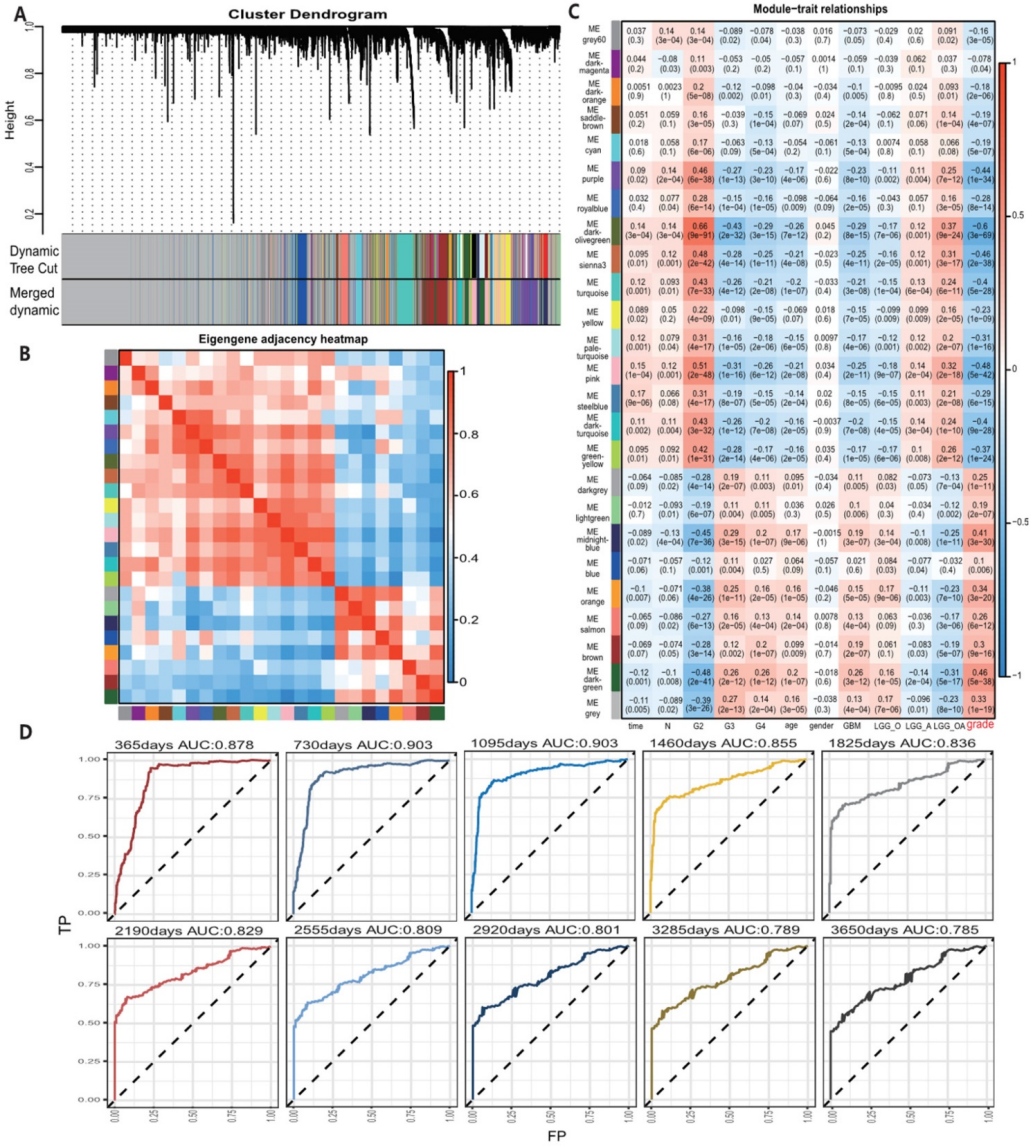
A, Module identification by WGCNA in the TCGA dataset and 24 gene modules were identified with statistical significance. B, Module correlation with correlation coefficient from 0 to 1, sienna3 and pink modules were positively correlated with darkolivegreen module. midnightblue and orange modules were positively correlated with darkgreen module. C, Module-trait correlation and the coefficient listed in the cells, positive correlation shows by color red and negative correlation in blue and p-value list in the brackets. Darkolivegreen module was the most significantly negatively correlated with tumor grades while darkgreen module was the most positively correlated with tumor grades. Sienna3, pink, orange, midnightblue modules had a strong correlation with tumor grades. D, Survival prediction ROC curve of prognosis model from 1 to 10 years and it showed 4 gene model possessed a high predictive power with AUC from 0.785-0.903.

A total of 24 modules were identified by WGCNA in TCGA gene expression profile data (Fig. 1A-C), of which the darkolivegreen module showed the most significant negative correlation with tumor grade (r = -0.6, p = 3e-69). The pink and sienna3 modules were also inversely correlated with the darkolivegreen module and tumor grade. (r = -0.48, P = 5e-42; r = -0.46, P = 2e-38), while the darkgreen module showed the most positive correlation with tumor grade (r = 0.46, P = 5e-38). The midnightblue and orange modules had positive correlation with the darkgreen module and tumor grade (r = 0.41, P = 3e-30; r = 0.34, P = 3e-20). We finally chose these 6 modules, which contained 705 genes in the TCGA cohort, for further study. Also, 12 modules in the meta_GSE1 dataset were identified by WGCNA (Figure S1A-C). Of these modules, the brown module showed the most significant negative correlation with tumor grade (r = -0.6, P = 6e-50), and the magenta module was highly correlated with the brown module and negatively correlated with tumor grade (r = -0.41, P = 2e-22) as the brown module. The red module had the most significantly positive correlation with tumor grade (r = 0.58, P = 1e-46). The blue and tan modules were highly correlated with the red module and had positive correlation with tumor grade (r = 0.5, P = 5e-34; r = 0.42, P = 6e-23). We then identified five modules associated with tumor grade, which contained 254 genes, in the meta_GSE1 cohort. Finally, we selected 67 intersected genes in these two cohorts as candidate glioma progression-associated genes for further analysis (Table S2).

### Construction of the prognostic gene signature

**Figure 2:**
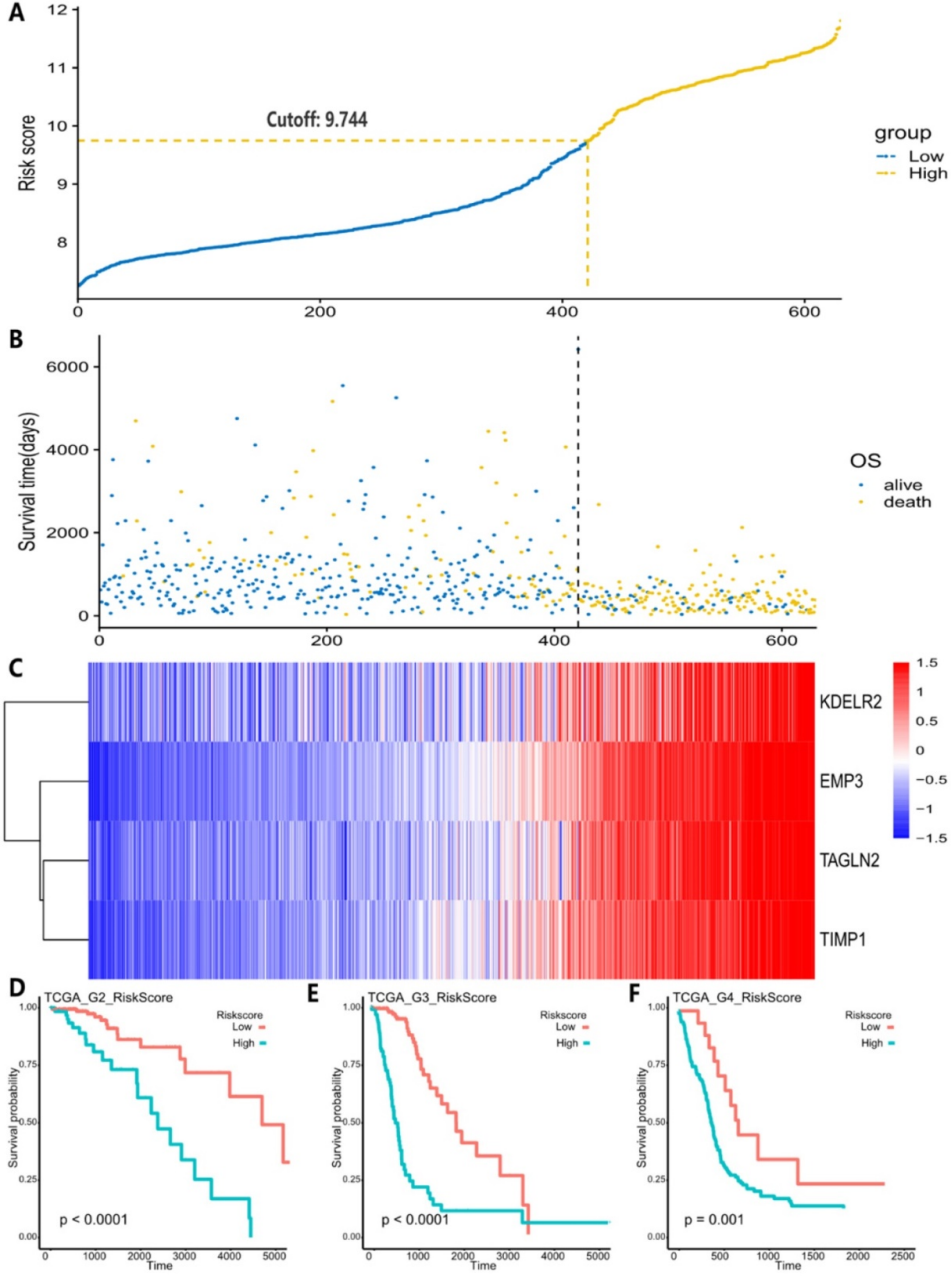
A, Risk score distribution and the optimal cut-off of TCGA dataset for distinguishing high- and low-risk groups. B, Survival distribution of TCGA datasets and its correlation with risk score. C, 4 genes' expression in TCGA dataset and its correlation with risk score. The heatmap sorted by risk score increasingly. D-F, different tumor grades' K-M curve and they showed that high-risk group and low-risk group had significant survival differences in TCGA data.

All 67 genes were statistically significant in the TCGA cohort (P<0.05) after the univariate Cox regression. We also performed the univariate Cox analysis of 67 genes in each pathological grade and 37 genes, which possessed statistical significance among all grades, were identified, suggesting that the prognostic role of these genes was independent of tumor pathological grades (Table S3). Next, we performed the LASSO regression analysis to build a robust model with these selected 37 genes. We first used 37 genes as an input for LASSO regression analysis. After 10^5^ times iterations, two stable models were obtained: one was a 5-gene model with the best predictive performance when minimum lambda was obtained (mod_37_5). The other was a 4-gene model that contained the least number of variables with ideal predictive efficiency and was called mod_37_4. A similar procedure was performed for 67 genes mentioned above and two models (mod_67_6 and mod_67_5) were obtained, including 6 and 5 genes in each model, respectively. We did not observe an obvious predictive power difference of four models through the three methods: C_index, CPE, and IDI (Table S4). Therefore, we chose the simplest model with good predictive power, mod_37_4 for further study. The risk score was derived from these 4 genes. ROC curves of survival prediction power from 1 to 10 years showed that the risk score had a high predictive power across different time points (AUC=0.785~0.903) (Fig. 1D). Multivariate Cox analysis showed that the risk score was an independent prognostic factor (HR=1.633, P = 9.695E-06) (Table 1). Then, we divided patients of all grades into high or low subgroups according to the risk score, for that the independent prognostic role of this score in all grades (Fig. 2).

### Validation of the prognostic gene signature

To further verify the model prediction power, we divided samples into the low- and high-risk groups according to the optimal cut point in five independent datasets which were not used for model building (CGGA_microarray, CGGA_RNA-Seq, GSE16011, GSE68848, GSE74187). And we observed a significant difference in survival amid all these datasets (Fig. S2-S6). The dataset GSE74187 contained only GBM samples and was considered as an independent validation set for GBM. The results showed that the risk score remained its distinction ability in survival differences (Fig. S6). The multivariate Cox model revealed that the risk score was an independent prognostic factor in all validation sets (Table S5). Besides, we found that all four genes were highly expressed in patients with a high-risk score.

### Pathway analysis based on prognosis model

**Figure 3:**
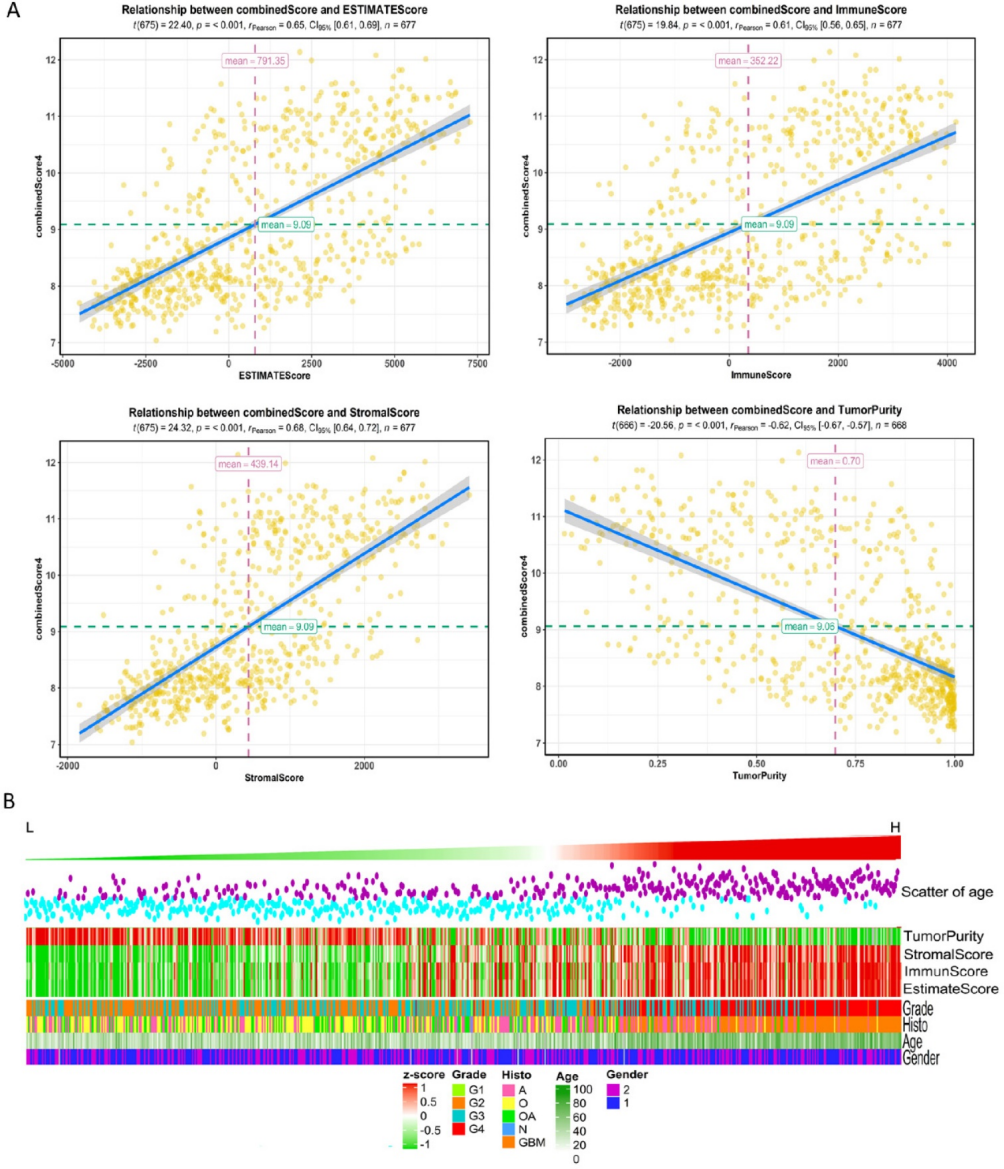
A, The correlation between risk score and ESTIMATE score, immune score, stromal score, and tumor purity in training datasets. B, High-risk group mainly consisted of GBM while low-risk group mainly comprised of LGG, including oligodendrogliomas (O), oligoastrocytomas (OA) and astrocytomas (A) histologically. Tumor purity, stromal score and immune score were normalized as z-score from -1 to 1.

**Figure 4:**
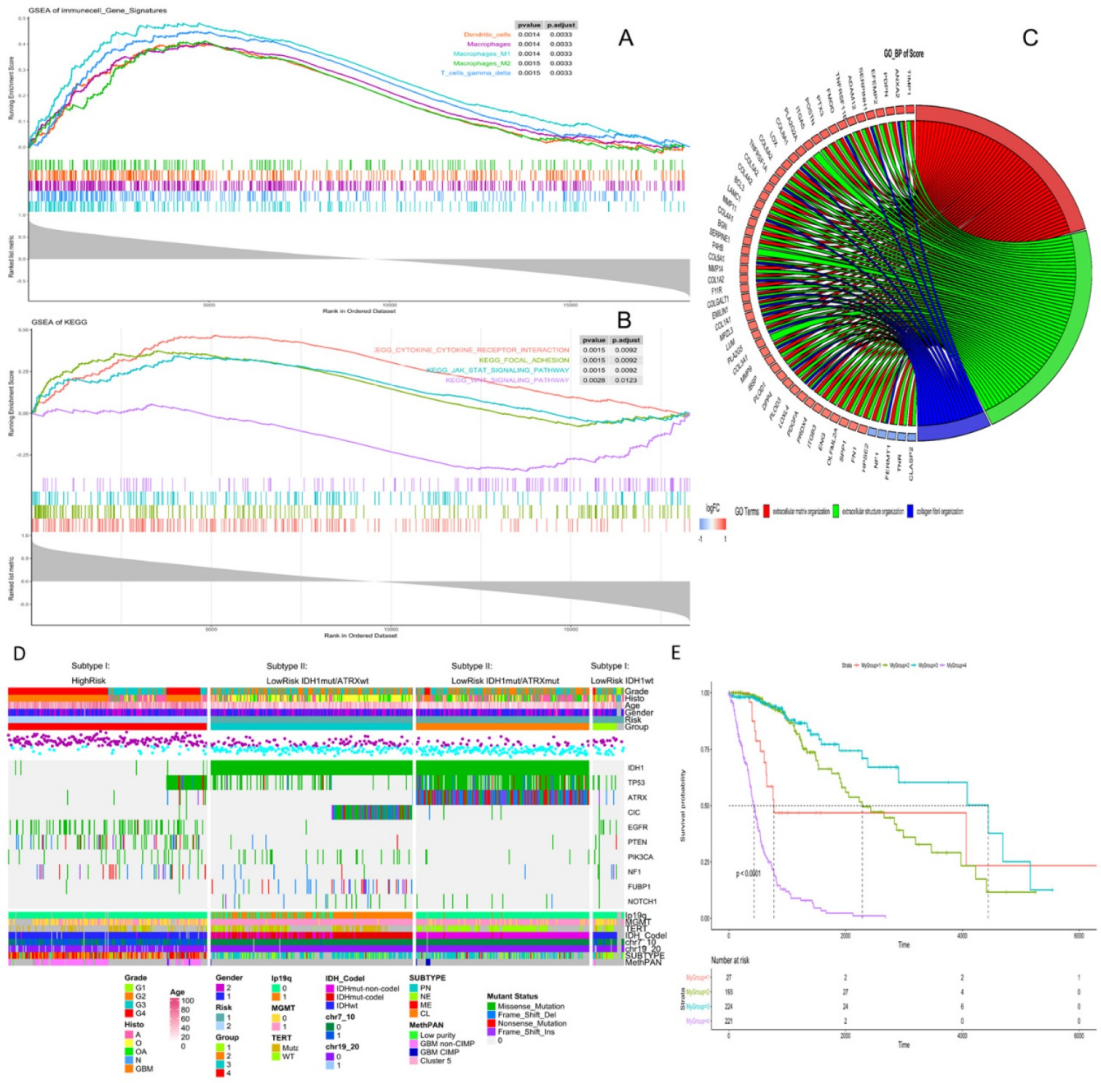
A, Identification of immune signals' difference between low-risk and high-risk groups by GSEA. Macrophages, Macrophages M1, Macrophages M2, Dendritic cells, T cells gamma delta, were activated in the high-risk group. B, Identification of risk score-related KEGG pathways by GSEA, and KEGG_CYTOKINE_CYTOKINE_RECEPTOR_INTERACTION, KEGG_FOCAL_ADHESION, KEGG_JAK_STAT_SIGNALING_PATHWAY were highly enriched in the high-risk patients. C, Identification of risk score-related GO pathways, extracellular matrix related pathways were gained. Genes in extracellular matrix organization pathway are in red, genes in extracellular structure organization are in green, genes in collagen fibril organization are in blue. D, Clinical, histological and genetic features between high- and low-risk groups. According to IDH mutation and ATRX mutation, the low-risk group could be divided into three subgroups, and each group had specific genetic characteristics, including glioma specific mutations, CNVs and Varhaak subtypes for GBM. E, K-M curve of four subgroups, group1- LowRisk_IDH1wt, group2- LowRisk_IDH1mut/ATRXmut, group3- LowRisk_IDH1mut/ATRXwt, group4-High risk.

High risk scores were closely related to ESTIMATE score, immune score, and stromal score, but negatively correlated with tumor purity (Fig. 3). We drew the GOplot for GO-annotation of genes that were significantly associated with risk scores (r ≥ 0.7). Finally, 83 GO BP-related terms enriched in the high-risk group were obtained (qvalue < 0.05), among which these genes mainly involved in cellular processes such as extracellular structure organization, collagen fibril organization (Fig. 4C). To further discover the underlying functional mechanisms that lead to different prognosis in patients between high- and low-risk groups, we used GSEA for gene expression analysis based on ImsignSignature and KEGG database. The results indicated that nine immune signals, such as Macrophages, Macrophages M1, Macrophages M2, Dendritic cells, T cells gamma delta, were activated in the high-risk group (Fig. 4A). KEGG analysis enriched 52 pathways significantly correlated with the risk scores, including KEGG_CYTOKINE_CYTOKINE_RECEPTOR_INTERACTION, KEGG_FOCAL_ADHESION, and KEGG_JAK_STAT_SIGNALING_PATHWAY, etc. which were highly enriched in the high-risk patients (Fig. 4B).

### Clinic features and genomic background based on prognosis model

After comparing the high-risk group with the low-risk group, we found no difference in gender between the two groups. The average age of patients in the low-risk group is 41.06±12.61y (n=455), which was lower than that in the high-risk group (58.63±12.97y, n=222) with statistical significance (t=-16.636, p-value<2.2e-16). As for the pathological grades, the high-risk group mainly consisted of high-grade gliomas, glioblastomas (GBM), while the low-risk group contained mostly LGG (p=1.17e-80) composed of oligodendrogliomas (O), oligoastrocytomas (OA), and astrocytomas (A) histologically (Fig. 3B). The risk score was associated with distinct genomic alterations. According to the glioma classification reported by Verhaak et al. 13, we discovered proneural subtype (PN) and neural subtype (NE) gliomas are predominantly involved in the low-risk group, and classical subtype (CL) and mesenchymal subtype (ME) gliomas accumulated predominantly in the high-risk group contrarily. Besides, G-CIMP- and G-CIMP+ gliomas enriched in the high-risk and the low-risk group separately. Loss of heterozygosity (LOH) at 1p/19q, IDH mutation, MGMT promoter methylation largely enriched in the low-risk group. On the other hand, the gain of chr 7/loss of chr 10 occurred in the high-risk group mainly (Table 2). We acquired consistent results after conducting the same analysis in the independent validation datasets (Table S6).

The risk level separated by the risk score reflected the multi-omics alteration: IDH1 mutation, the most common mutation in glioma, was involved mainly in the low-risk group. In our exploration, we found out that by combining IDH1 mutation with ATRX mutation, gliomas in the low-risk group could be further divided into three subtypes: LowRisk_IDH1^wt^, LowRisk_IDH1^mut^/ATRX^mut^ and LowRisk_IDH1^mut^/ATRX^wt^ with obvious survival difference (Fig. 4E). LowRisk_IDH1^wt^ group contained A, O, OA, and GBM without 1p/19q codeletion. LowRisk_IDH1^mut^/ATRX^mut^ group contained more A with a high mutation frequency of TP53 and few 1p/19q codeletions. LowRisk_IDH1^mut^/ATRX^wt^ group, consisted of O largely, enriched almost all gliomas with 1p/19q codeletion, CIC mutation, and FUBP1 mutation. However, IDH1wt and mutation of EGFR, PTEN, NF1 and RB1 were mostly enriched in the high-risk group (Fig. 4D, S7C). According to the overview of the mutation landscape, we could find that TP53 mutation existed in all subtypes. The mutation of EGFR and IDH would not coexist in the same sample and the same relationship appeared in ATRX mutation and TERT mutation. Besides, we discovered that TERT mutation mostly exists in LowRisk_IDH1^mut^/ATRX^wt^ group and the high-risk group, and CIC mutation accompany with 1p/19q codeletion.

Then the differential methylation analysis of high-grade and low-grade gliomas was performed on 62,813 methylation regions, of which 47,271 regions were significantly correlated with the risk score (r>0.5, p<1.0E-15) and were widely distributed on all chromosomes. Among them, 46,116 (97.6%) regions were negatively correlated with the risk score and 1155 (2.4%) regions had a positive correlation. These negative-correlated methylations, which accumulated in LowRisk_IDH1mut/ATRXmut and LowRisk_IDH1mut/ATRXwt subtypes of the low-risk group, were mainly associated with IDH1 mutation. Meanwhile, 1155 positive-correlated methylation regions were mainly associated with IDH wild type (Fig. S7A). We further explored the degree of methylation of 154 probes that could further distinguish patients of the high-risk group into two subgroups with significant differences in overall survival (Table S7).

The CNV-changing genes that were significantly associated with risk scores were mainly enriched on chr1, 7, 10, and 19. The gene deletion on chr1 was mainly enriched in LowRisk_IDH1mut/ATRXwt subgroup; while chr7 was mainly amplified in all grades of gliomas, especially in the high-risk group. In IDH1 wild-type gliomas, chr10 deletion coexisted with chr7. Chr19 was mainly characterized by the deletion in LowRisk_IDH1mut/ATRXmut and LowRisk_IDH1mut/ATRXwt subgroups, especially in the latter subgroup, and its amplification predominantly in the high-risk group. The frequency of changes in chr19 in LowRisk_IDH1mut/ATRXmut subgroup was significantly lower than that of chr1, indicating that the co-deletion of chr1 and chr19 occurred only in LowRisk_IDH1mut/ATRXwt subgroup (Fig. S7B). We further found that the 314 genes, as risk factors, significantly affected the overall survival rate of patients with LowRisk_IDH1mut/ATRXmut subtype gliomas. These genes were located on chromosomes 9p22 and 10q24. And the amplification of 212 genes, locating at chromosomes 8p21 and 7q21, significantly affected the overall survival rate of patients with LowRisk_IDH1mut/ATRXwt subtype gliomas (Table S8).

## Discussion

All four genes were positively correlated with tumor pathological grades. Epithelial Membrane Protein 3 (EMP3), a 4-transmembrane glycoprotein, is identified firstly as a putative tumor suppressor in gliomas 14. Jun F et al. 15 found that EMP3 is highly expressed in CD44-high primary GBMs, and promote tumor progression by TGF-β/Smad2/3 signaling pathway activation. Recent studies 16 showed that EMP3 expression, whose high-level expression is identified as a prognostic indicator of poor survival, is significantly higher in high-grade gliomas than in low-grade gliomas or normal brain tissues. KDELR2, a transmembrane protein of the endoplasmic reticulum, could recognize proteins with KDEL (lam-aspartate-glut-leucine) tetrapeptide sequence, then mediating these proteins' recycling from the Golgi back to the endoplasmic reticulum. It has been found that KDELR2 stimulates ECM degradation by inducing Src activation at the invadopodia and leads to phosphorylation of the Src substrates, cortactin, thereby promoting tumor metastasis and invasive growth 17. The expression level of KDELR2 in GBM is significantly higher than that of LGG and could be used as a prognostic marker for overall survival 6. TAGLN2, an actin-binding protein, could regulate cell morphology, movement and transformation by binding to actin. TAGLN2 is abnormally expressed in a variety of cancers 18, and its deregulation is considered to correlate with tumorigenesis and tumor development. In gliomas, TAGLN2 is a potential oncogenic factor, which is regulated by TGFβ2 to promote glioma invasion and growth 19. Its expression, particularly enriched in mesenchymal subtype of glioma, is associated with pathological grades and poor prognosis. TIMP1 belongs to the matrix metalloproteinase (MMP) family and inhibits most MMPs, especially selectively inhibits MMP9, participating in the homeostatic regulation of the extracellular matrix. TIMP1 plays an important role in the IDH wild type gliomas, which could promote the survival of cancer cells by negatively regulating the adaptive immune response 20. It has also been reported that glioblastoma patients with lower expression of TIMP-1get longer survival 21. Aaberg-Jessen et al. 22 found that co-expression of TIMP-1 and CD63 have effects in glioblastoma stemness and contribute to the poor prognosis of patients through influencing tumor aggressiveness and resistance of therapy. These above evidences reveal the prognostic value of the four genes for glioma patients which could further be used as potential drug targets or gene candidates in predicting patients' prognosis.

GSEA of the 4-gene signature showed that macrophages, dendritic cells and gamma delta T cells activities related to patients' high-risk scores. Also, KEGG CYTOKINE CYTOKINE RECEPTOR INTERACTION, KEGG JAK-STAT SIGNALING PATHWAY, KEGG FOCAL ADHESION were significantly enriched. Macrophages/microglial cells constitute the largest immune cell populations in GBMs 23. These cells promote tumor proliferation, invasion, and maintenance 24. The cytokines, secreted by immune cells or stromal cells (such as vascular endothelial cells, epidermal cells, and fibroblasts), participate in the immune response and inflammatory process, regulating cell proliferation, differentiation, and apoptosis. JAK/STAT is a common pathway for many cytokine-signaling 25. Cell adhesion is essential for interaction with the extracellular matrix (ECM) during tumor cells invasion 26. The percentage of ECM components and inflammatory immune response in the microenvironment is associated with tumor purity. The progress of glioma is accompanied by angiogenesis and invasiveness, which inevitably depends on the production of a large number of adhesion molecules and extracellular matrices 27. The low level of ECM and adhesion molecules in tumor means high tumor purity, and patients with high tumor purity usually commit low-grade gliomas, IDHmut glioma, or proneural GBM with better clinical outcomes 28. In contrast, low-purity tumors have higher cell adhesion and extracellular matrix content, mainly found in IDHwt and mesenchymal subtype GBM and associated with poor prognosis 29. Meanwhile, these low-purity gliomas are enriched with immunological processes and inflammation 30. Thus, we applied the ESTIMATE algorithm 31 to predict tumor purity using gene expression profiles. It showed a significant positive relationship of ESTIMATE score, immune score, stromal score, low tumor purity (an indicator of poor prognosis in glioma 28) and high-risk score.

The clinical features of glioma are also important factors that influence the prognosis of patients. We found that the risk scores of older patients were higher than the youth, which means a worse prognosis in older patients, and the result was consistent with the existing research conclusions 32. Compared with the low-risk group, the high-risk group was mainly composed of GBM and lower-grade glioma (G2/G3) with poor prognosis. This group mainly contained the currently known molecular and genetic characteristics that contribute to poor prognosis, such as CL and ME subtypes 13, non-LOH at 1p/19q, IDH1wt, G-CIMP- and MGMT promoter unmethylation 33, chr7 gain and chr10 loss 29. We also found that a small number of GBM patients with a relatively good prognosis can be divided into the low-risk group by this scoring system. To further understand the underlying molecular mechanisms of risk score, we used TCGA data to analyze the association of gene mutations, methylation, CNV and risk score. We found that patients in high and low-risk groups had significant differences in gene mutation characteristics. Even within the same risk group, the patients in the low-risk group could be divided into three subtypes with different overall survival prognosis based on IDH1 and ATRX mutation. ATRX mutation is mutually exclusive with 1p/19q codeletion 34 and 1p/19q codeletion often represents a better prognosis. Methylation analysis revealed that methylation probes, negative-correlated with risk scores, were enriched in the low-risk group and correlated with IDH1 mutations. On the contrary, positive-correlated methylation probes were primarily associated with IDH wild type glioma. We found that CNV-changing genes that significantly associated with risk scores were mainly enriched on chr1, 7, 10, and 19, and each risk group had specific CNV characteristics respectively. In our study, the risk score was correlated with the current major glioma molecular characteristics, and through combining methylation and CNV features this model can be used to further subdivide gliomas into different subtypes. We also found that the deletion of genes on 9p22/10q24 and 8p21/7q21 affected the prognosis of low-risk group patients with IDH mutations, which suggests that amplification or deletion of these chromosomal fragments may play a potential role in specific LGG subtypes. Simultaneously, partial methylation probes possess similar ability to stratify the patients in high-risk groups. All these deserve further study.

However, several limitations of our study should be taken into consideration. The incompleteness of follow-up data, no progress-free survival analysis was performed, and no more datasets were further validated for the re-stratification of the four subtypes (CL, PN, NE, ME). Second, all data were based on microarray and sequencing expression data, so their predictive effects were only applicable to mRNA expression and not to protein expression levels. Third, the prognostic model only considered genes that were highly expressed in lower-grade glioma relative to adjacent cancer but did not consider the prognostic role of non-differentiated genes. Some molecules might not be highly expressed in cancer, but still affected the prognosis of patients via other means. Furthermore, the molecular mechanisms of how the 4-gene signature affected the prognosis of glioma patients should be further elucidated by a series of experiments.

## Conclusion

In summary, we established a malignant-related 4-gene molecular marker by glioma expression profile data from multiple microarrays and sequencing data. The four markers had good predictive power on the overall survival of glioma patients and were independent of pathological grades which could be potential treating targets for improving glioma patients' prognosis. To our knowledge, the 4-gene signature-related integrated multi-omics prognostic model has not been reported previously and could be a useful prognostic and diagnostic classification tool of diffuse gliomas.

## Supplementary Material

Supplementary figures.

Supplementary tables.

## Figures and Tables

**Table 1:** Multivariate Cox multivariate analysis

Factor	HR	Lower_CI	Upper_CI	p value
age	1.0214	1.0062	1.0369	0.005816
gender	0.9167	0.6707	1.2530	0.585471
hist_GBM	0.4834	0.2059	1.1351	0.095105
hist_LGG_O	0.6788	0.3695	1.2469	0.211736
grade	2.6161	1.5405	4.4426	0.000372
hist_LGG_A	1.0190	0.5849	1.7753	0.946989
hist_LGG_OA	1.0000	NA	NA	NA
KPS	0.9834	0.9712	0.9957	0.008511
RiskScore	1.6327	1.3139	2.0287	**9.69E-06**

**Table 2:** Distribution overview of risk groups

Characteristics	N	L_risk	H_risk	X-squared	p-value
**Gender**				1.055	0.304
Male	386	252	134		
Female	284	197	87		
**Grade**				368.1	1.17E-80
Grade II	248	238	10		
Grade III	263	201	62		
Grade IV	161	11	150		
**Histology**				367.084	2.98E-79
A	192	141	51		
GBM	161	11	150		
O	190	179	11		
OA	128	118	10		
**IDH_STATUS**				527.552	9.64E-117
WT	238	27	211		
MUT	425	420	5		
**IDH_1P19Q_SUBTYPE**				106.61	5.42E-25
noncodel	498	281	217		
codel	168	168	0		
**MGMT_PROMOTER_STATUS**				169.203	1.10E-38
nonMethy	164	49	115		
Methy	474	399	75		
**CHR7_GAIN_CHR10_LOSS**				354.11	5.40E-79
WT	507	438	69		
MUT	156	8	148		
**TERT_PROMOTER_STATUS**				49.614	1.87E-12
WT	163	155	8		
MUT	157	98	59		
**ATRX_STATUS**				101.316	7.84E-24
WT	463	259	204		
MUT	195	188	7		
**DNAMethyl_PANCAN**					1.11E-07*
GBM non-CIMP	109	2	107		
GBM CIMP	9	7	2		
Cluster 5	3	0	3		
Low purity	6	0	6		
**TRANSCRIPTOME_SUBTYPE**				354.038	1.99E-76
PN	241	224	17		
NE	114	99	15		
CL	88	4	84		
ME	102	14	88		

*Fisher's Exact Test.

## Abbreviations

DGs: diffuse gliomas
LGG: lower-grade gliomas
GBM: glioblastomas
IDH: isocitrate dehydrogenase
MGMT: O6methylguanine DNA methyltransferase
TERT: telomerase reverse transcriptase
CNV: copy number variation
TCGA: the cancer genome atlas
CGGA: Chinese glioma genome atlas
TOM: topological overlap matrix
MEs: module eigengenes
CPE: Concordance Probability Estimate
IDI: integrated discrimination improvement
ROC: receiver operating characteristic curve
KEGG: Kyoto Encyclopedia of Genes and Genomes
GO: Gene Ontology
GSEA: gene set enrichment analysis
O: oligodendrogliomas
OA: oligoastrocytomas
A: astrocytomas
PN: proneural subtype
NE: neural subtype
CL: classical subtype
ME: mesenchymal subtype
G-CIMP: Glioma CpG island methylator phenotype
LOH: loss of heterozygosity
ATRX: X - linked alpha thalassemia mental retardation syndrome gene
MMP: the matrix metalloproteinase
ECM: extracellular matrix
