# Supplementary material for: Systematic Analysis of 4-gene Prognostic Signature in Patients with Diffuse Gliomas Based on Gene Expression Profiles
Source: J Cancer. 2021 May 19;12(14):4295–306. doi: 10.7150/jca.54565 (PMC8176424; doi:10.7150/jca.54565)
Supplement: Supplementary file 1 — Supplementary figures. [file jcav12p4295s1.pdf]

## Supplementary Figures

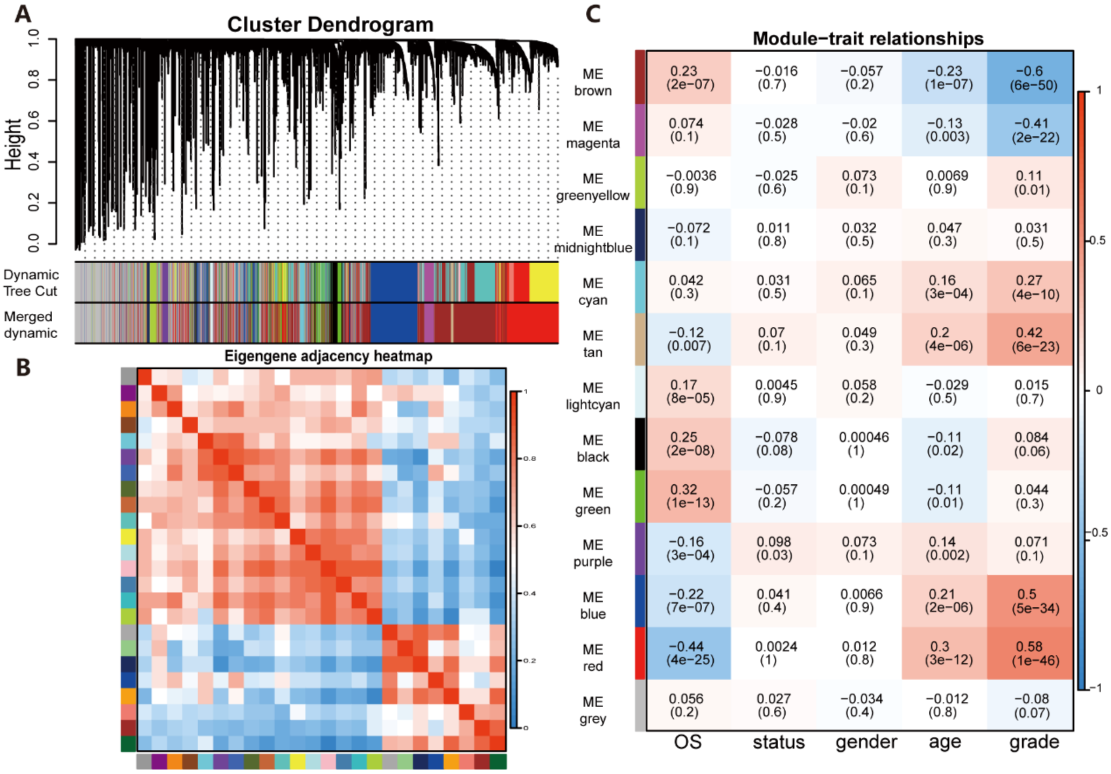


Figure S1, A, Module identification by WGCNA in the GSE-meta1 dataset and 12 gene modules were identified. B, Module correlation with clinical traits, magenta module was positively correlated with brown module. blue and tan modules were positively correlated with red module. C, Module-trait correlation, brown module was the most significantly negatively correlated with tumor grades while red module was the most positively correlated with tumor grades. Magenta, blue and tan modules had a strong correlation with tumor grades, positive correlation shows in red and negative correlation in blue, p values list in the brackets.

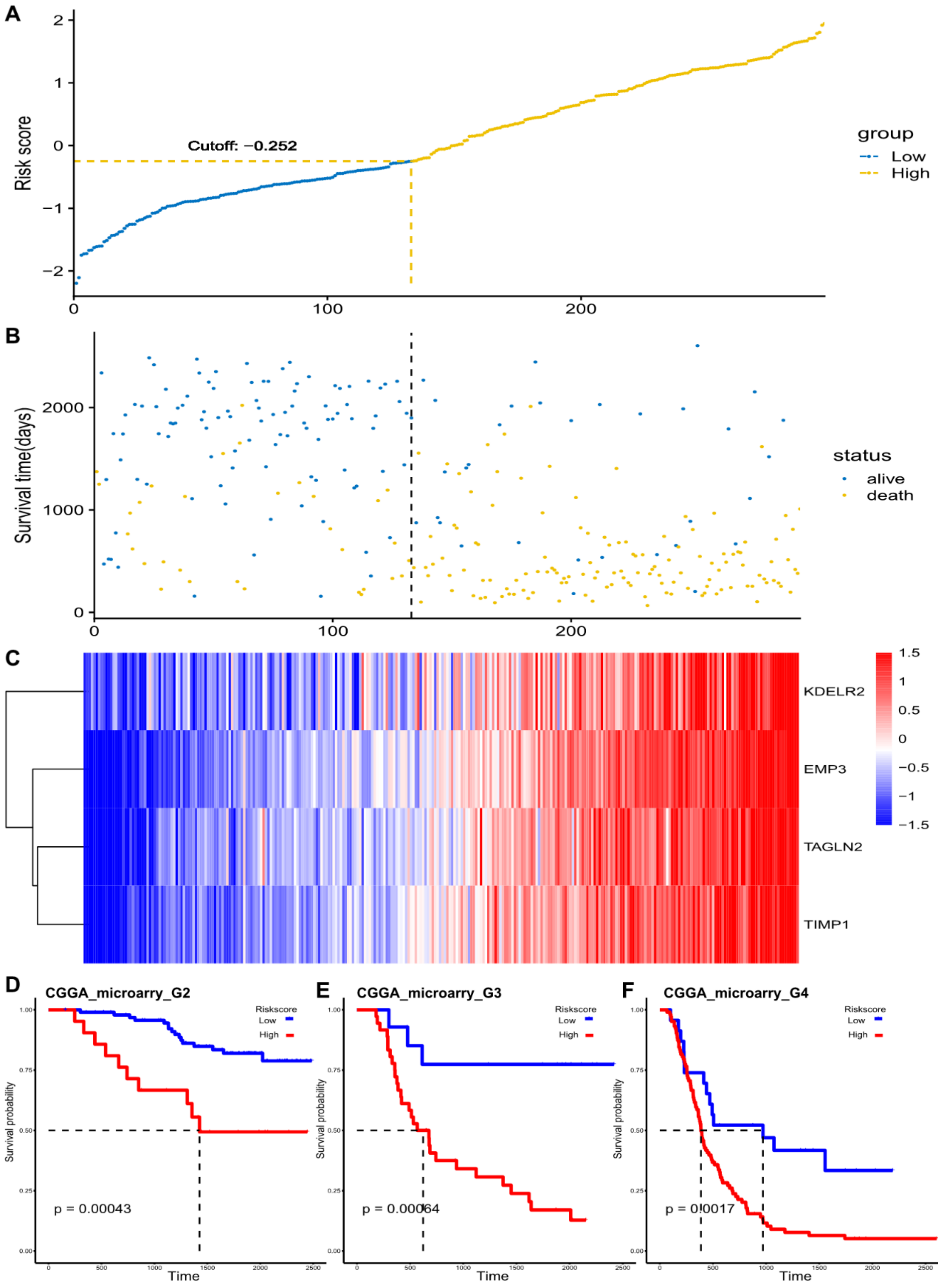


Figure S2, A, Risk score distribution and the optimal cut-off of CGGA microarray data for distinguishing high- and low-risk groups. B, Survival distribution and its correlation with risk score. C, 4 genes' expression and its correlation with risk score, the heatmap sorted by risk score increasingly. D – F, different tumor grades' K-M curve and they showed that high-risk group and low-risk group had significant survival differences in CGGA microarray data.

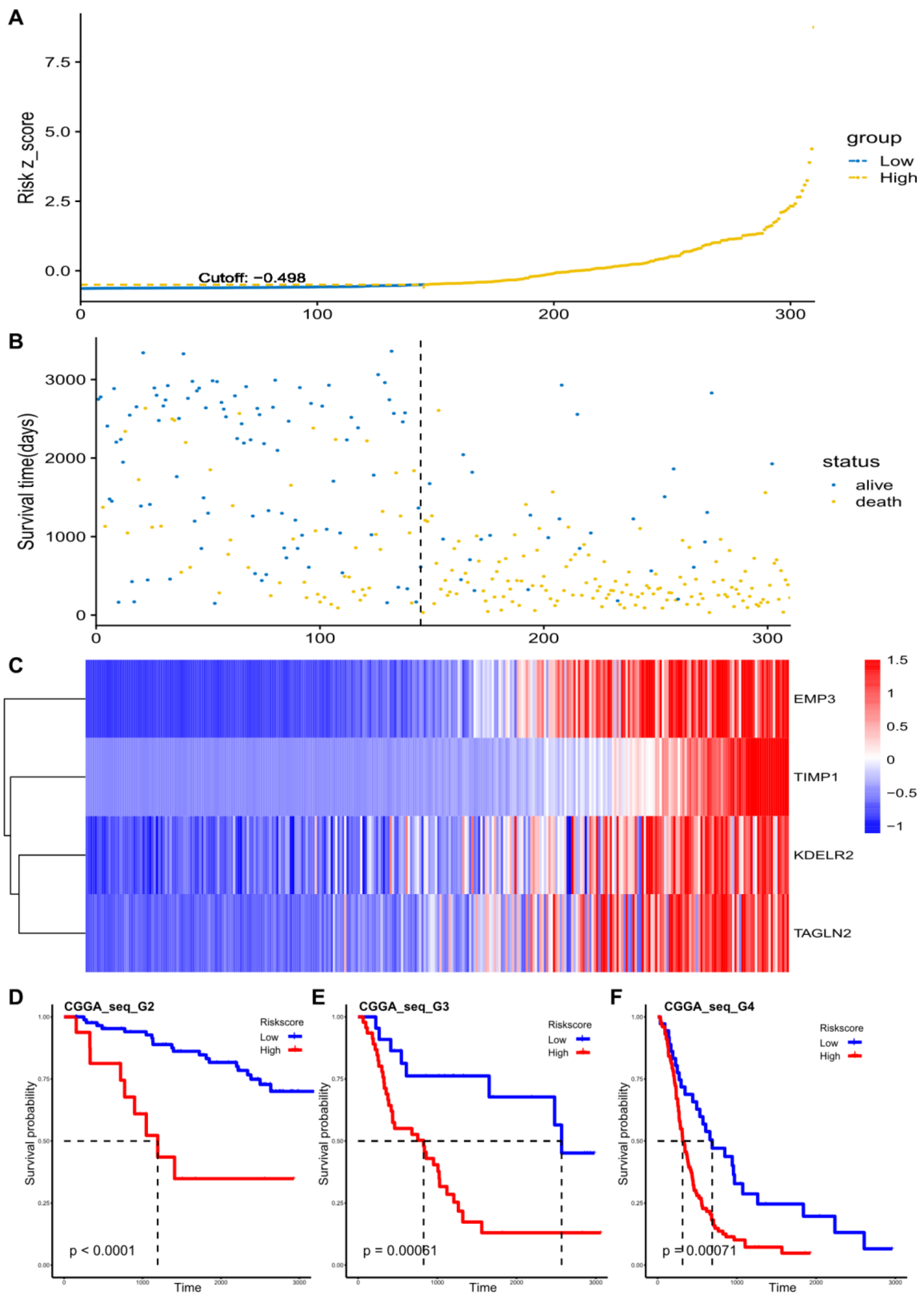


Figure S3, A, Risk score distribution and the optimal cut-off of CGGA RNA-seq data for distinguishing high- and low-risk groups. B, Survival distribution and its correlation with risk score of CGGA RNA-seq data. C, 4 genes' expression and its correlation with risk score, the heatmap sorted by risk score increasingly. D – F, different tumor grades' K-M curve and they showed that high-risk group and low-risk group had significant survival differences in CGGA RNA-seq data.

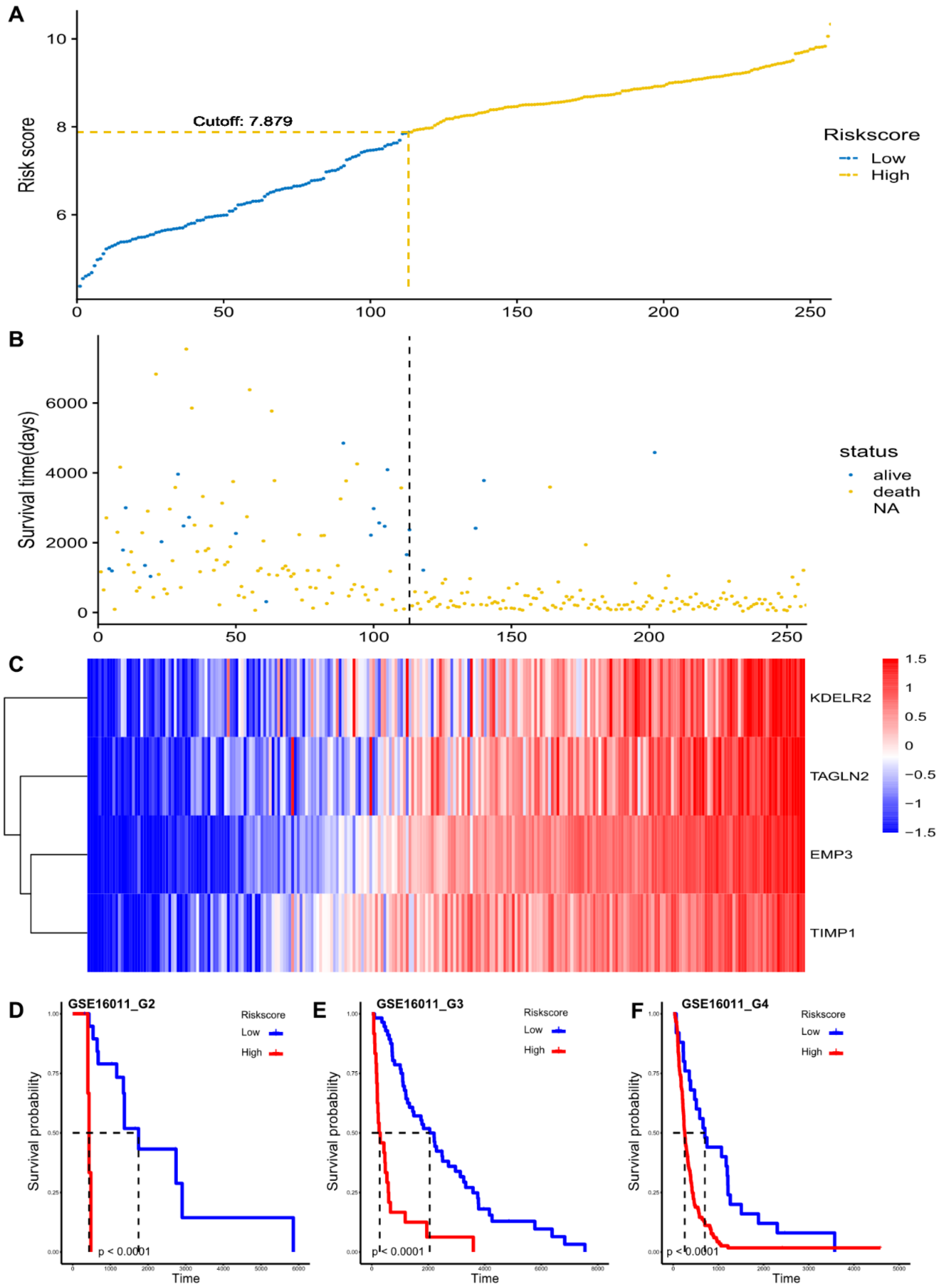


Figure S4, A, Risk score distribution and the optimal cut-off of GSE16011 data for distinguishing high- and low-risk groups. B, Survival distribution and its correlation with risk score of GSE16011. C, 4 genes' expression and its correlation with risk score, the heatmap sorted by risk score increasingly. D – F, different tumor grades' K-M curve and they showed that high-risk group and low-risk group had significant survival differences in GSE16011 data.

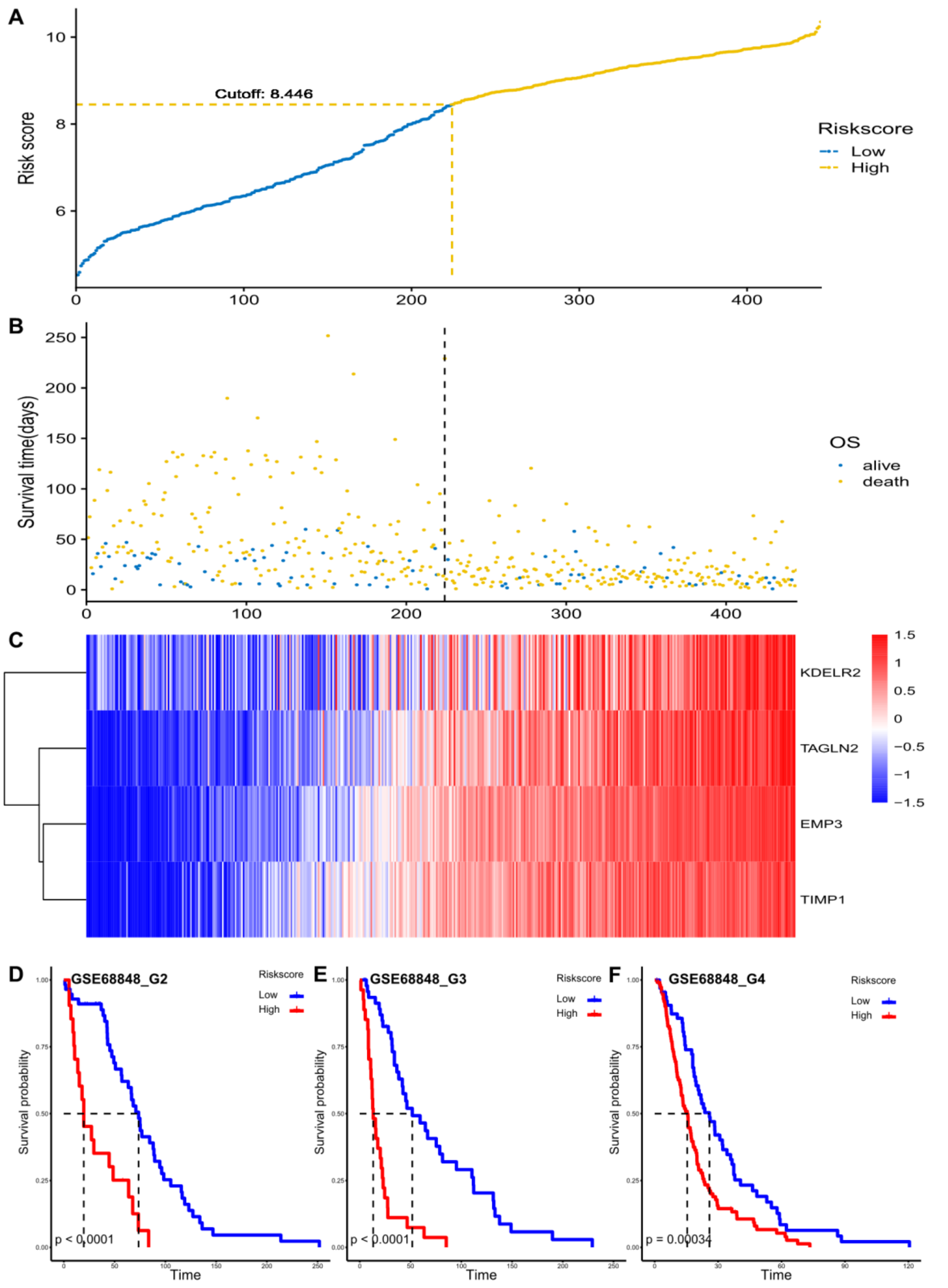


Figure S5, A, Risk score distribution and the optimal cut-off of GSE68848 data for distinguishing high- and low-risk groups. B, Survival distribution and its correlation with risk score of GSE68848. C, 4 genes' expression and its correlation with risk score, the heatmap sorted by risk score increasingly. D – F, different tumor grades' K-M curve and they showed that high-risk group and low-risk group had significant survival differences in GSE68848 data.

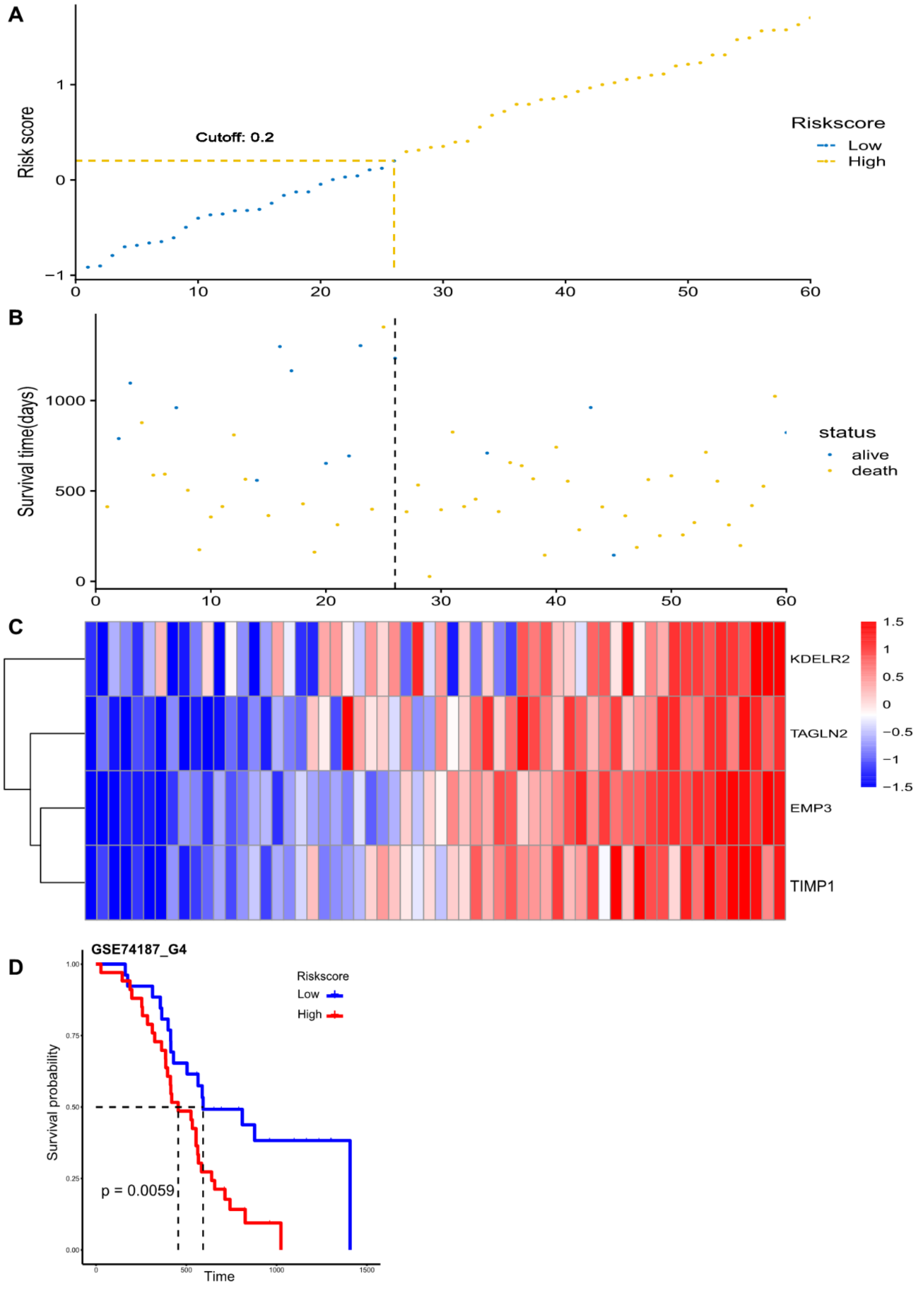


Figure S6, A, Risk score distribution and the optimal cut-off of GSE74187 data for distinguishing high- and low-risk groups. B, Survival distribution and its correlation with risk score of GSE74187. C, 4 genes' expression and its correlation with risk score, the heatmap sorted by risk score increasingly. D, GBMs' K-M curve and it showed that high-risk group and low-risk group had significant survival differences in GSE74187 data.

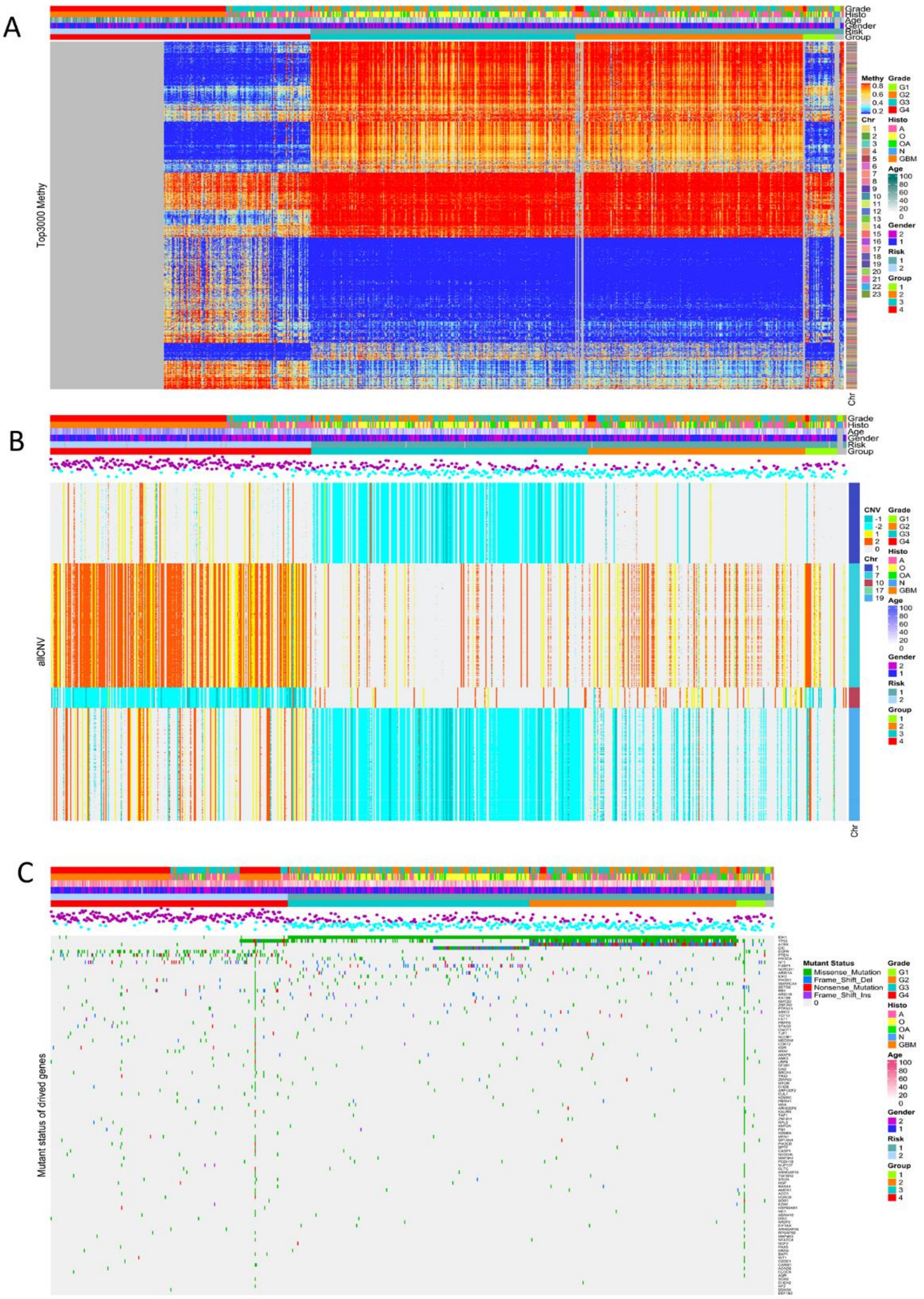


Figure S7, A, methylation difference in high-risk group and each subgroup of low-risk group, the samples without methylation data were shown in grey part. B, CNV difference in high-risk group and each subgroup of low-risk group, red and yellow means insertion and blue means deletion. C, Mutation difference in high-risk group and each subgroup of low-risk group. Group1- LowRisk_IDH1wt, group2-LowRisk_IDH1mut/ATRXmut, group3- LowRisk_IDH1mut/ATRXwt, group4-High risk.
